# Lipids from Insects in Cosmetics and for Personal Care Products

**DOI:** 10.3390/insects13010041

**Published:** 2021-12-30

**Authors:** Antonio Franco, Rosanna Salvia, Carmen Scieuzo, Eric Schmitt, Antonella Russo, Patrizia Falabella

**Affiliations:** 1 Department of Sciences, University of Basilicata, Via dell’Ateneo Lucano 10, 85100 Potenza, Italy; antonio.franco@unibas.it (A.F.); carmen.scieuzo@unibas.it (C.S.); 2 Spinoff XFlies s.r.l., University of Basilicata, Via dell’Ateneo Lucano 10, 85100 Potenza, Italy; 3 Protix B.V., Industriestaat 3, 5107 NC Dongen, The Netherlands; Eric.Schmitt@protix.eu; 4 Greenswitch s.r.l., Strada Provinciale Ferrandina—Macchia, 75013 Ferrandina, Italy; antonella.russo@greenswitch.it

**Keywords:** beauty-care products, bioconversion, black soldier fly, circular economy, fatty acids

## Abstract

**Simple Summary:**

The use of insects as a new source of lipids is a topic of great interest from both environmental and economic points of view. In addition to use in feed and energy applications, lipids could be used for the formulation of personal care products. The cosmetics industry is always in search of new ingredients to use in novel product formulations. The processes mediated by bioconverter insects, such as *Hermetia illucens*, are really advantageous because starting from substrates of low economic and biological value (agri-food by-products, zootechnical, catering, and other waste), it is possible to obtain products of high commercial value. The composition of insect lipids depends on the feeding substrate, as well as the insect species, therefore for each personal care application, it is possible to find the most suitable starting conditions. In this review, we display a general outlook on insect lipids, the extraction processes, and their use in cosmetics and personal care fields.

**Abstract:**

Insects, the most varied group of known organisms on Earth, are arousing great interest also for the possibility to use them as a feed and food source. The mass rearing of some species, defined as “bioconverters”, is spreading worldwide, thanks to their sustainability. At the end of the bioconversion process, breeders obtain eco-friendly biomolecules of high biological and economic value, including proteins and lipids, from larvae of bioconverter insects, in particular *Hermetia illucens*. Besides the most classical use of insect lipids as food additives, they are also used in the formulation of several products for personal care. The composition of insect lipids depends on the substrate on which the insects are reared but also on the insect species, so the cosmetic producers should consider these features to choose their insect starting point. The most abundant fatty acids detected in *H. illucens* are lauric, myristic, palmitic, and oleic acids, regardless of feed substrate; its fatty acids composition is favorable for soap composition, while their derivatives are used for detergent and shampoo. Here, we offer an overview of insect lipids, their extraction methods, and their application in cosmetics and personal care products.

## 1. Introduction

### 1.1. Insects

Insects (Arthropoda: *Insecta*) are not only the most specious group of known organisms on Earth, but they also represent the greatest example of biodiversity. To date, over a million species have been documented and many more await discovery [1,2,3]. Insect biodiversity reflects the extraordinary ability to colonize all geographical regions and ecological areas, because of their small size, adaptability, aerial mobility, dispersal, and phenotypic plasticity [4]. To survive in extreme living conditions, to escape and tolerate environmental challenges, this class of invertebrates has evolved three different types of adaptations: physiological, behavioral, and morphological [5]. Thanatosis, migration, changes in body colors and patterns (crypsis and mimicry), accumulation and synthesis of cryoprotectants (sorbitol, glycerol), which reduce the lethal freezing temperature of the body, and changes in lipid membrane composition, are examples of different kinds of insect adaptations [5]. Although all organisms in the ecosystem are essential, the role played by insects is particularly crucial for maintaining ecosystem functions and providing remarkable ecosystem services vital to humans [6]. Moreover, with their activity, insects impact the global economy too; consequently, the preservation of these organisms and their diversity is a topic of worldwide interest [2].

**Figure 1:**
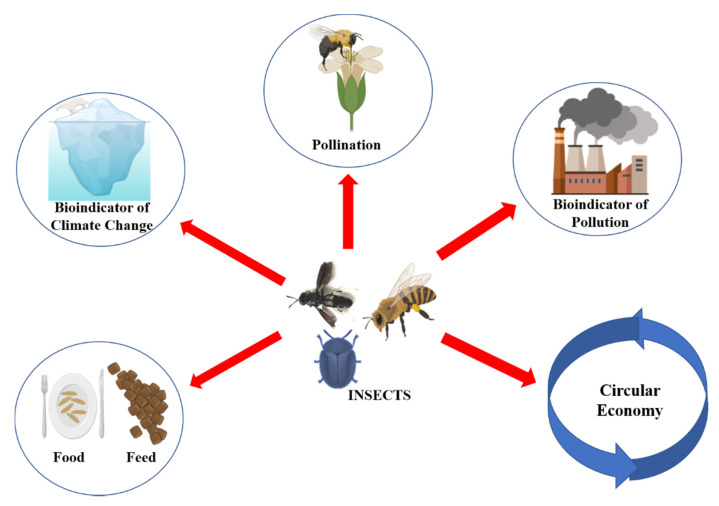
Insects play a key role in ecosystem functions maintenance. They are the key pollination agents of most crops and wild vegetation and find a role in the circular economy. Moreover, they are bioindicators of climate change and pollution and find applications in feed and food.

In addition to their role as natural/biological pest controller and chief agent of pollination for most fruits and other agricultural products, insects are important bioindicators of climate change, crop management, aquatic and land environment quality, and, therefore, they act as decisive environmental pollution indicators (Figure 1) [7]. As decomposers, they promote the release of nutrients from animal and vegetable matter, helping in nutrient recycling, soil turnover, improving water and carbon storage, and stimulating vegetal growth and diversity [8,9]. Moreover, some of them are great bioconverters, able to actively contribute to the realization of a circular economy with a perspective on zero-waste production [10]. Their bioconversion process works on different kinds of feeding substrates, from vegetal to animal matter [11,12]; this is one of the best features that make these insects so useful and sustainable (Figure 1). Among the bioconverter insects, some species are considered of particular interest for feed and prospectively for food. Although insect farming for food is still at early stages, some species have been successfully raised on a medium-large scale. Indeed, according to European Regulations 873/2017 and 2021/1372, seven insect species (*Hermetia illucens*, *Musca domestica*, *Tenebrio molitor*, *Alphitobius diaperinus*, *Acheta domesticus*, *Gryllodes sigillatus*, and *Gryllus assimilis*) are allowed for aquaculture, poultry, and pig farming, if reared on substrates from the agri-food sector [13,14]. Although only recently *T. molitor* was approved for human consumption by the European Food Safety Authority (EFSA) [15], edible insects have the potential to become the future feed and food due to their beneficial nutritional characteristics and low environmental impact. Many studies, indeed, reported the life cycle assessments of insect rearing, demonstrating a lower global warming potential estimation compared to conventional animal farms and composting methodologies [16,17,18]. The profitability of edible insects is different according to different species, typologies of products, and different European and global markets [19]. The rearing of insects on by-products and waste from different industries reduces the cost of feed thanks to the circularity of the process. The global insect feed market will reach a value of USD 1.4 billion by 2024 [20]. Insect farms have raised €600 million for investments in insect production through September 2019, according to the International Platform for Food and Feed (IPIFF); moreover, the European production of insect protein each year is about 6000 tonnes, but the production could reach up to five million tonnes by 2030 [21]. Most bug farms are based on small-scale production selling their products in niche markets as specialty items [22], even if there are some large farms based on an industrial scale [23].

### 1.2. Lipids from Insects

Insects store lipids for two reasons: firstly, as an energy reserve for the adult stage which often has reduced or absent nutrition activity [24]; secondly, lipids allow insects to reduce transpiration and store non-imbibed water [25]. Lipid reservoir modulates several important aspects of the insect’s life, such as the timing of metamorphosis, egg development, rate of insect growth, behavior, and reproduction regulation in the adult stage [26,27]. Indeed, intermediary metabolism in insects and the exchange of metabolites take place in fat body tissues [26,27,28]. These factors and regulations are managed by a controlling network of multiple signals such as hormonal, nutritional, and environmental signals that also regulate the insect fat body biology [27,29]. Lipids are the second-largest nutritional fraction of insects, and their content is highest in the larval stage of insects’ life [30], varying from 10 to 50% on a dry basis [30,31]. The composition of insect lipid and fatty acid profiles are influenced by several factors, such as species, life stage, diet, environmental conditions, migratory flight, and sex [18,30]. In most cases, female insects contain more lipids than males [32]. Indeed, Bentz reported that female adults of the Mountain Pine Beetle (*Dendroctonus ponderosae*) contain a higher lipid content than male adults [33]. On the other hand, Gilbert et al. described that in male adult moths (*Cecropia moths*) the lipid content was much higher than in the female specimen (21.6 vs. 5% per gram of wet weight) [34]. Additionally, Jackson et al. reported that wild-type males of *Drosophila melanogaster*, contain more surface lipids than female exemplars, but with a little difference (3.5 ± 0.2 vs. 3.0 ± 0.1 mg surface lipid/g of body weight) [35]. Sex differences in lipid composition are related to differences in gender nutritional requirements for optimal performance and maximization of fitness, which are known to be sex-specific [36]. For female reproduction, adult fat body tissues play crucial roles, especially synthesizing and secreting vitellogenin and other required proteins [27]. The main reserve site for insect lipids is fat body, and the lipid composition of the internal organs probably reflects its composition [37]. In response to the energy demand, lipids stored in this organ are degraded, processed, and then transported to the site of employment [38]. Lipids are also constituents of the insect cuticle, helping to keep water in the body and contributing to communication between populations of social insects and within insects of the same sex [38,39,40]. Some functions of the body fat are predominantly localized in certain regions while other functions are present all over the tissue [41,42]. In most insects, lipid stores accumulation commonly increases in diapause or overwintering stages [43,44] since their lipids are the principal fuel for overwintering and post-winter activities [45]. In Table 1, examples of lipid yield and fatty acid composition of the seven insect species that could be reared for feed production for aquaculture, poultry, and pig farming [13,14] are reported. Among these species, *H. illucens*, also known as black soldier fly (BSF), has both the highest and the lowest lipid content reaching 40.7 ± 1.9% on a dry matter basis for larval biomass reared on fruit waste and only 8.1 ± 0.9% in the case of larvae reared on brown algae [46]. *T. molitor* showed the second-highest lipid yield (31.97 ± 1.60%/DW) while the lipid content of the other species ranges from 15.31% of *A. domesticus* to 26.25% of *A. diaperinus* [47,48,49,50,51]. The wide range of lipid content of BSF larvae (BSFL) could be easily explained by the ethology of this stage, where they can feed on several organic substrates with different compositions which influences the final larval composition. Concerning the fatty acid composition, only BSFL showed a considerable amount of lauric acid (C12:0), the most suitable for cosmetic applications (23.40–61.87% of total fatty acids). Additionally, the diet composition also affects the resulting fatty acid composition and percentage: prepupae fed on fermented maize stover had the lowest amount of lauric acid, while prepupae reared on vegetable and fruit waste have the highest percentage [46]. In all the analyzed species, the palmitic acid (C16:0) covers up to 20% of the fatty acid profile, except for *M. domestica* which showed the lowest yield (5.10 ± 0.07%) and *A. diaperinus* with the highest (24.98 ± 0.05%). Myristic acid (C14:0) was mostly detected in BSFL (3.8–10.4%) reared on vegetable and fruit waste, and in *T. molitor* (4.45 ± 0.02%), although in lower concentrations; for other species, the percentage was almost irrelevant. Finally, oleic acid (C18:1 n-9) was detected in quite high concentrations (20.18–35.83%) for all the species except for BSFL reared on vegetable and fruit waste (4.27%) and for *M. domestica* (7.21 ± 0.08%). In Table 1, coconut oil, palm oil, and kernel palm oil are also reported to make a comparison with insect fatty acid composition. Generally, these vegetable sources contain more lipid content than analyzed insects; only BSFL, when reared on fruit waste, gave a similar result of commercial palm oil and commercial palm kernel oil (42.64 vs. 45.00–55.00 and 50.00%/DW, respectively). Lauric acid was the most concentrated fatty acid detected both in coconut oil and commercial palm kernel oil (44.10–51.00 vs. 47.80%) and BSFL reared on vegetable and fruit waste (61.87%) [52,53,54,55]. Meanwhile, for commercial palm oil, the dominant fatty acids were palmitic and oleic acids (44.00% and 39.20%) [54,56]. In these cases, commercial palm oil has the best lipid yields compared with all the analyzed species.

## 2. Extraction of Insect Lipids

Different methodologies for lipids extraction are currently available, as reported in Franco et al., [46], that can be applied also to insect lipids. Tzompa-Sosa et al. [57] compared three extraction procedures: the Folch method, aqueous extraction, and the Soxhlet method. The aim of Tzompa-Sosa and colleagues was to evaluate the lipid yield from different insect species of economic interest, that in Europe are allowed to be reared for feed production for aquaculture, poultry, and pig farming: *H. illucens*, *T. molitor*, *M. domestica*, *A. domesticus*, *A. diaperinus*, *G. sigillatus*, and *G. assimilis* [47,50,51,57,58,59,60,61]. Besides the conventional fat extraction methods (Folch and Soxhlet extractions), the authors used the aqueous extraction described by Yi et al. [62]. Table 2 reports the lipid yield for each of the seven insect species after different extraction methods. For all the analyzed species, the most used and high-performing method is extraction by the Soxhlet apparatus. Among the various solvents that can be used for the above-mentioned extraction, petroleum ether was the most used, but ethanol gave the best lipid yield in *T. molitor* (28.80 ± 5.90%/WW) and *A. domesticus* (22.70 ± 2.90%/DW) [58]. In all samples, the aqueous extraction, being a green extraction without the use of chemical solvent, gave the worst result in terms of lipid content (1.50–8.20%/WW); indeed, this methodology allows to obtain mainly non-polar lipids and lipids with a lower ω-6/ω-3 ratio than the Folch and Soxhlet extractions [57].

In the study of Laroche et al., three lipid extraction methods were compared (conventional extraction by Soxhlet with solvents, three-phase partitioning (TPP), and extraction with supercritical carbon dioxide fluid (SC-CO_2_) [58]. Lipid yield and their fatty acid profiles were evaluated on *A. domesticus* and *T. molitor*. The extractive yields were higher for *T. molitor* (21.50–34.70%/WW) than *A. domesticus* (11.90–25.60%/WW) [49]. For *A. domesticus*, a higher impact of the extraction method on the lipid yield was recorded. Particularly, significant differences were observed for the extractions with the Soxhlet method in relation to the solvent used. The extraction with ethanol produced the highest yield (22.7 ± 2.90%/WW). The lipid yields with TTP were similar to those obtained with ethyl acetate or ethanol. The yields obtained by extraction with hexane and SC-CO_2_ were the lowest (14.60 ± 0.10% and 11.9 ± 1.40%, on wet weight respectively). The yield with the SC-CO_2_ method could be implemented using a co-solvent, as shown in the study by Rudyk and colleagues for the extraction of non-polar and non-food compounds [63]. No significant differences were documented for lipid extraction from *T. molitor* with the six different methodologies. The in-depth analysis in fatty acid profile showed that the three most abundant fatty acids detected were palmitic acid, vaccenic acid (C18: 1V), and linoleic acid (C18: 2 (n-6)), for both *A. domesticus* and *T. molitor*. For *A. domesticus*, linoleic acid represents 29.00 –35.80% of the total, while palmitic acid and vaccenic acid have percentages of 23.90–29.9% and 19.00–22.90% respectively. Stearic acid (C18: 0) is the fourth most abundant fatty acid in *A. domesticus* with a percentage of 9.20–10.60% of the total fatty acids. For *T. molitor*, the vaccenic acid is the most abundant fatty acid (36.94–40.10% of the total fatty acids). The second most abundant was linoleic acid (33.30–37.10%), followed by palmitic acid (17.73–19.30%) [58]. Except for the described fatty acids, the remaining lipids have been detected in small quantities. Additionally, Purschke and colleagues have studied the supercritical fluid extraction method from *T. molitor* larvae [64]. In their study, the influence of the extraction conditions, the degreasing performance, and the extracted lipid composition were evaluated, comparing them with a cold extraction method using hexane (C_6_H_14_) as a solvent. The extraction with hexane was carried out using sodium sulfate (Na_2_SO_4_) and hexane. The fat residue extracted was 21.10 ± 0.35% on a dry basis. This yield was lower than others reported in literature that ranged from 31.10% to 43.08% [65,66]. These differences in the yield values may depend on the diet administered to insects before lipid extraction [11,65], and the particle size of the ground insect samples [67,68]. The chemical-physical properties and the composition of the lipids extracted with SC-CO_2_ fluid were similar to that obtained with extraction with hexane. The data obtained by Purschke and colleagues [64] are comparable to those obtained by Tzompa-Sosa and colleagues [57] for *T. Molitor*. In both studies, the degree of degreasing with the Folch/Soxhlet methods was almost 100% (96.56 ± 0.39 and 98.40 ± 0.24%, respectively), and aqueous extraction was 60.30 ± 0.40%. The composition of the extracted lipids was significantly affected by the extraction parameters. The maximum degreasing degree (95%) with SC-CO_2_ liquid extraction was reached at 400/250 bar, 45 °C, and 105 min. Extracting with hexane at the same conditions, the degreasing degree was 96.56 ± 0.39%. At lower pressure, the free fatty acids content of the lipids extracted with SC-CO_2_ significantly increased, with a maximum of 8.64 ± 0.46% obtained applying 250 bar, 65 °C for 45 min, since the solubility of triacylglycerols in SC-CO_2_ fluid decreases with the reduction of the density of the solvent. For this reason, the focus was shifted towards free fatty acids which have better solubility in SC-CO_2_ fluid than the tri-, di-, and monoglycerides. Anyway, it is necessary to apply an industrial method of lipid extraction from insects to maximize the yield and production of these valuable molecules. Currently, there are commercially available oil extraction machines whose specific use is for lipids extraction from *H. illucens*. The BSFL dried biomass is pressed to obtain fat and partially defatted meal. During the process, there are two products obtained: the fat from larvae that squeezes out and a press cake (partially defatted meal with high protein content). Besides the BSFL protein powder that is yielded by grinding the press cake after filtration processes, BSFL fat is obtained by the press liquid. Moreover, the production of protein meal and fats of BSFL using a screw press can be obtained with two methods: wet processing and dry processing. Wet processing involves fresh larvae that are directly pressed. Press cake needs further drying and grinding to reduce its moisture content and particle size and the separation of the press liquid is more difficult, and advanced equipment like a centrifuge would be needed. Instead, dry processing involves a drying process of BSFL before pressing. The press cake may be ground to BSFL meal directly and the BSFL fat can be refined by a filtration or decanting step. Up to 60% of the mass of crude BSFL fat is composed of solids that, during the fat refining, are separated from the fat. The processing time for fresh larvae takes more time even if the screw press works in the same way for both wet and dry processing. Compared with larvae dried with a classic oven, the larvae dried with microwave are easier to press. Wet larvae are heavier, but the main problem is that the extraction produces not only fats; indeed, also water is released, increasing the pressing time [69]. Anyway, currently, there is no scientific evidence for this method about extraction yield and lipids characterization.

Generally, the extraction of insect lipids is related to subsequent biodiesel production. For example, the production of 1 kg of lipids from dried BSFL, compared to 1 kg of lipids from rapeseed caused an increase of 0.2 kg CO_2_-eq (global warming potential) and 9.8 MJ (energy use), mainly related to the food waste transport and larval drying process and a decrease of 6.44 m^2^/annual (land use) [70]. Lipid extraction from larvae results in a high environmental impact, but it is fundamental to take into account that the usage of land for the production of conventional sources of lipid (sunflower, rapeseed, etc.) is no more sustainable, while the rearing of insects on by-products has the advantage of disposing of this waste and obtaining several products of interest, including lipids.

## 3. Lipids in Cosmetics and Personal Care Products

Fats and oils are commonly used in the cosmetic industry for skincare, and they are the major components of body care creams. In particular, we refer to insect oil when insect lipids are liquid (at room temperature) or insect fat if insect lipids are in a solid phase [71]. The fatty acids and their derivatives, commonly used as components of cosmetic products, are a mixture of triglycerides of saturated and unsaturated fatty acids, together with synthetic esters, fatty alcohols, silicones, etc. Fatty acid soaps are commonly used as emollients for softening the skin. Indirectly, they hydrate the skin by reducing trans-epidermal water loss [72]. The properties of fats can differ depending on the fatty acid profile. For instance, the healing or protective properties of the creams can be intensified using linoleic acid for dry skin, which decreases loss of water from open burn wounds and helps in the protection of the skin against damage and drying out [73,74,75,76]. Moreover, fats are used in cosmetic products for their emulsifying properties and for contributing to the increase of formulation viscosity. Unsaturated fatty acids strengthen the skin barrier function, prevent moisture loss, provide for structural integrity damaged by external agents, and have anti-inflammatory properties [77]. However, some sources of lipids have ethical problems. For example, although mink oil is considered a secondary derivative of the industrial processing of animal fur, there were considerable ethical objections regarding the use of animals to produce fur [78], and, for this reason, this source of fats has not been used in cosmetics for many years. There is, therefore, a need to find alternative lipid sources. Macadamia nut oil could be, for example, an alternative source suitable for cosmetics [79]. Despite these considerations, some disputes may arise due to the use of valuable land for non-food use, the transport costs, and the release of pollutants throughout the process. Therefore, the identification of more suitable sources for cosmetic applications becomes fundamental. Insects could provide a promising solution for this application; lipids from insects can be used as a possible alternative to vegetable oil, normally used for the preparation of products for skincare and protection. Therefore, they have also an application in the cosmetic industry as natural ingredients of cosmetic formulations.

Below are some of the main fatty acids and their derivatives from insects, used in reported cosmetic applications.

### 3.1. Lauric Acid (C12:0)

**Figure 2:**
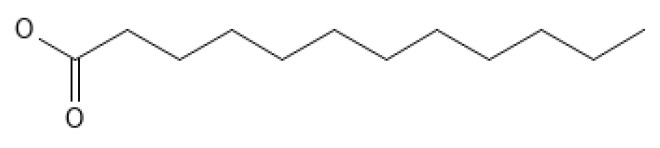
Lauric acid chemical structure, obtained with Draw Structure software by PubChem (https://pubchem.ncbi.nlm.nih.gov/#draw=true (accessed on 8 October 2021)). Myristic and palmitic acids have similar structures to lauric acid, differences are related to side-chain lengths that are composed of 14 and 16 carbons, respectively.

Coconut and palm kernel oils (about 50%) are the main sources of lauric acid (Figure 2); a lower percentage is represented by babassu butter (approximately 40%), with other vegetable oils, such as camphor seed oil, and milk fats [80,81,82] and its chemical synthesis is described by numerous patents [80]. Lauric acid is produced by the hydrolysis (mostly via saponification) of vegetable and animal fats and oils, followed by a fractional distillation [83,84]. Lauric acid occurs in various forms: white or slightly yellow powder or glossy crystalline [80,81,83] or as a colorless solid [80]. In the cosmetic field, lauric acid derivatives (esters, ethoxylated compounds, etc.) behave as surfactant cleansing agents, commonly called surface-active agents; they are ingredients that help two substances that normally do not mix to become dissolved or dispersed in another one. Substantially, they clean skin and hair by helping water to mix with oil and dirt so that they can be rinsed away. According to SpecialChem–the material selection platform [84], lauric acid is currently contained in 92 products for personal care.

### 3.2. Myristic Acid (C14:0)

The main sources of myristic acid (Figure 2) are coconut oil, nutmeg butter (*Myristica fragrans* Houtt), palm seed oils, and milk fats [80,81]. Up to 80% of myristic acid is contained in seed oils of Myristicaceae, but a lower percentage has been found in most vegetable oils and animal fats [85]. Commercial myristic acid is produced by the saponification and fractionation of vegetable and animal fats and oils [85] and it is nontoxic when ingested [86]. Myristic acid occurs as a hard, white, or faintly yellow, glossy crystalline solid, as a white or yellow-white powder [81], or as colorless leaflets [82]. The composition of myristic acid is tetradecanoic acid (95% minimum), hexadecanoic acid (4% maximum), and dodecanoic acid (3% maximum) [84]. Myristic acid is also composed of unsaponifiable material such as hydrocarbons, at a maximum concentration of 0.2%; however, some grades may contain glyceryl monomyristate at a maximum concentration of 0.07%. Butylated hydroxytoluene may also be present as an added antioxidant [85]. According to Becker and colleagues, myristic acid and its derivatives are safe as cosmetic ingredients [87]. According to SpecialChem—the material selection platform [84], myristic acid derivatives are currently contained in 45 products for personal care.

### 3.3. Palmitic Acid (C16:0)

The main sources of palmitic acid (Figure 2) are Chinese vegetable tallow (60–70%) [80,81], palm oil (30–50%), lard and tallow (25–30%), cocoa butter (25%), and other kinds of vegetable butter in a minor percentage. Generally, commercial palmitic acid is mixed with variable amounts of stearic acid; palmitic acid is a mixture of solid organic acids obtained from fats, both vegetable and animal. It appears hard, white or dimly yellow, faintly glossy crystalline solid to a white or light-yellow powder [82], white crystalline [80], or colorless crystals [83]. Palmitic acid is produced by the hydrolysis and fractionation of vegetable and animal fat. Usually, the fractionation is made by distillation or crystallization [80,83,87]. According to SpecialChem—the material selection platform [84], palmitic acid derivatives are currently contained in 255 products for personal care.

### 3.4. Oleic Acid (C18:1 n-9)

**Figure 3:**
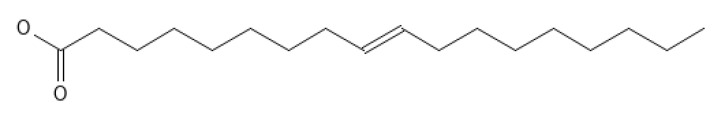
Oleic acid chemical structure, obtained with Draw Structure software by PubChem (https://pubchem.ncbi.nlm.nih.gov/#draw=true (accessed on 8 October 2021)).

Generally, oleic acid (Figure 3) constitutes more than 50% of the total fatty acid concentration in many vegetable oils and animal fats. Olive oil (80%), peanut oil (60%), teased oil (85%), and pecan oil (85%) are very rich in oleic acid. It is improbable to find an oil with less than 10% of this acid [81]. At 4 °C, it solidifies to a crystalline mass. At temperatures higher than 5–7 °C, oleic acid occurs as a colorless to soft yellow, oily liquid. The exposure to oxygen creates a gradual darkening of the compound while it is subjected to a decomposition process if heated to 80–100 °C, at atmospheric pressure [80,83,88]. The two processes involved in oleic acid production are hydrolysis and fractionation through saponification and distillation of vegetable and animal fats and oils [80,83,84,89]. Oleic acid is commercially used in the form of substances combined with 7–12% saturated acids and some unsaturated acids. It is derived from edible sources only, such as olive oil and rapeseed oil. Oleic acid must be derived from edible sources [89]. According to SpecialChem—the material selection platform [85], oleic acid derivatives are currently contained in 367 products for personal care.

## 4. Insects as Lipids Source for Cosmetic Applications

The production of insect lipids has been evaluated on several species and *H. illucens* seems to be one of the most promising thanks to its composition and lipids yield. Indeed, the lipid content in BSFL and prepupae is about 15–49% of the total dry weight. Food substrates can affect the larval body composition, in terms of both protein and fat content [62,63,90], but the fatty acid composition of the food substrate did not affect the larval fatty acid composition [11]. The main macronutrient in the food substrate that affects the content of fats in BSFL are the carbohydrates [91,92]; it occurs because larvae can convert carbohydrates into fats [91]. In any case, the lipid content in BSFL is higher than other insects, fish meal, and soybean (9.9%/DM vs. 2.0%/DM) [93,94]. The lipid structure of BSFL fat is composed of a mix of saturated and unsaturated fatty acids and triglycerides [62,95]. Between all the lipid components, fatty acids and their derivatives are the most valuable. To test the feeding substrate impact on fatty acids composition, Surendra and colleagues tested prepupae fed on a mix of organic waste [96]: consistent with the results obtained by other authors [93,95,97,98,99], the study showed that the concentration of lauric acid and palmitic acid (short-chain saturated fatty acids) was 67% of total fatty acids, higher than coconut oil and palm kernel oil (55–57% of total fatty acids) (Table 1). On the other hand, the research even showed that the concentration of unsaturated fatty acids (28% of total fatty acids) was higher than in coconut oil (10%) and higher than in palm kernel oil (18%) (Table 1) [96]. For the other identified categories of fatty acids, further studies have affirmed that linoleic acid and alpha-linolenic acid (C18:3 (n-3)) were the main represented polyunsaturated n-6 and polyunsaturated n-3 fatty acids respectively, while oleic acid was the main represented monounsaturated fatty acids in BSFL [11,100,101,102]. Lauric acid is the most abundant fatty acid of the lipid fraction of BSFL [11,62,99,103]. Nowadays, generally, it is extracted from coconut oil and palm kernel oil. For their ability to create a foaming soap but also for its antimicrobial properties, lauric acid derivatives are a primary component for the preparation of soaps [104,105,106,107]. Lauric acid obtained from alternative sources, such as insects, with less environmental impact may replace the common sources (palm kernel oil, coconut oil).

The abundant presence of lauric acid could explain a hypothetical anti-bacterial activity of BSFL lipids because it is probably converted into monolaurin. Indeed, the latter is an antibacterial, antiviral, and antiprotozoal glyceride for animals and humans [108]. Among the main fatty acids that compose the lipid structure of BSFL, lauric acid and its monoglyceride derivative, monolaurin, show the strongest antimicrobial activity [109]. For the above-mentioned reasons, Verheyen and colleagues evaluated the possibility of using fats from three insect species (BSF, *Locusta migratoria*, and *A. domesticus*) for cosmetic formulations [79]. Fats extracted by the Soxhlet method using petroleum ether were used in different percentages (1%, 2%, 4%, 5%, and 10%) for the preparation of hand cream formulations. Different concentrations of fats were tested to evaluate the sensorial properties and physical stability of creams as a function of fat concentration. In Verheyen et al. paper, these formulations were compared with those containing 5% mink and macadamia nut oils. The results indicated that insects can be an alternative source to provide derivatives of such fatty acids that are suitable for the formulation of shower gel and soaps and their application depends on the fatty acid profile. Generally, despite a low amount of palmitoleic acid (C16:1 (n-7)) that is considered a great ingredient for good skin penetration, locust and cricket lipids are more suitable in cosmetics [79]. On the other hand, since the BSFL fatty acid profile is similar to that of palm kernel and coconut oil, its lipids are suitable for shower gels and soaps [79] (Table 1).

Fats extracted from these species were rich in some contaminants such as phospholipids and free fatty acids which had to be removed by extensive refining processes to make them more suitable for cosmetic applications [79]. This purification procedure, which involves the elimination of these contaminants from the raw extract that could reduce the shelf life is called “degumming” [110] and is used to neutralize free fatty acids. In addition to degumming, the refining process of the raw fats included neutralization, bleaching [111], and deodorization treatments [112]. The fat formulations obtained by Verheyen and colleagues, were incubated for about two months and physical/chemical parameters were monitored during the incubation period [79]. Colour, aroma, viscosity, pH, and general appearance were recorded at regular intervals. It was noted that, as lipid concentration was increased, the color of the cream became dark or greenish, depending on the extraction method. This phenomenon was particularly evident in samples with 10% insect fat, which resulted in hand creams with undesirable organoleptic properties. BSFL fats were rich in relatively short-chain fatty acids (C 12:0 and C 14:0), with a lower degree of unsaturation (21%), compared to that of locust and cricket fats (61%). Therefore, this analysis revealed that locust and domestic cricket fats were more suitable for use in cosmetics, because of their high amounts of saturated and unsaturated C18 fatty acids, comparable with the macadamia nut oils [79]. Examples of C18 chain fatty acids are linoleic acid (C18:2) and linolenic acid (C18:3), which reduce trans-epidermal water loss and regenerate the lipid barrier of the epidermis [113]. Since they contain high amounts of lauric acid [39,99,103,114,115], BSFL fats are not the best choice as ingredients for leave-on products, because they could be the cause of adverse effects on the skin lipid structure, disrupting the skin barrier and increasing the trans-epidermal water loss [116]. On the other hand, the lipid profile of BSFL is more similar to that of palm kernel oil and coconut oil [79,117] with a lauric acid content > 60%, resulting to be more suitable for cleaning products such as soaps and shower gels [79]. Overall, these results suggest that insect fats (especially cricket and locust fats) are suitable for leave-on cosmetic formulations, at least from a physicochemical point of view, and that BSFL fats can be used for the preparation of surfactants because of the high content of lauric acid [79]. The lauric acid, which is the most abundant of the total fatty acids in BSFL, confers several beneficial properties to larvae fat as a biologically active molecule [118]. Therefore, BSF larval fat could be used as an alternative preservative. Moreover, lauric acid and other fatty acids derivatives that compose BSF larval lipid fractions are important stabilizers of dispersed systems and emulsifiers and are used in the production of cosmetics and soaps [118,119]. Indeed, not only lauric acid but also palmitic acid (used as emollient and emulsifier), oleic acid (activating lipid metabolism), myristic acid, linoleic acid, and omega-6 fatty acids and their derivatives show notable properties and functional effects, contributing to the skin barrier, and are frequently used in the cosmetic industry [119].

Hence, BSFL is a source of bioactive compounds (fatty acid derivatives) that can be used as natural cosmetic ingredients, considering the quantity and high quality of fatty acids identified in the lipid composition of BSFL biomass.

Verheyen and colleagues demonstrated that BSFL fats could be a promising and suitable alternative to palm kernel or coconut oil for the synthesis of glycine-acyl surfactants, which can be used for technical applications [79]. Recently, researchers are focusing on amino acid-based surfactants since they can be produced using renewable sources, are biodegradable, less toxic than the traditional surfactants, and consequently, are safer for the environment. Sodium Cocoyl Glycinate is an example of a glycine-surfactant, commercially available, recommended for use in soap and facial wash products, which is produced from coconut oil. Verheyen and co-workers extracted fats from three different sources (palm kernel oil, coconut oil, and BSFL) and used them for conversion into glycine-acyl surfactants using the Schotten-Baumann reaction, in which a fatty acid chloride reacts with the amino group of glycine giving rise to surfactant. This procedure efficiently produced surfactant molecules which were subsequently characterized in terms of solubility, foaming capacity, and surface tension, and compared with the commercially available Sodium Cocoyl Glycinate. All these glycine-acyl surfactants showed similar properties to each other and revealed some differences with the commercial surfactant regarding the solubility and foaming properties. However, this study highlighted the possibility to produce surfactants using BSFL fats with satisfactory yields, comparable to that of commercial ones.

Recently, a skincare product produced using purified BSF larval fat has been patented and is internationally marketed [120]. This product shows several beneficial characteristics that improve skin conditions, including smoothing, revitalizing, moisturizing, and tightening the skin. Sangduan described the entire process necessary for the preparation of skincare products using BSFL as a source of fats [120]. Specifically, after BSFL drying, fats must be extracted by solvent extraction, a screw press, or SC-CO_2_ extraction, and separated from the rest of the mixture by filtration or centrifugation; then, the extract fats have to be sterilized and mixed with specific ingredients (such as vitamins), giving rise to a product with good appearance, good stability, and a good adsorption property. Therefore, BSFL are a promising source of different natural bioactive compounds which can contribute to skincare and can find wide application in cosmetic fields. BSFL can be a source also of other useful molecules that can be applied in cosmetics as well, such as antimicrobial peptides (AMPs). AMPs, which are small bioactive molecules with strong activity against several microorganisms, can be extracted from BSF, that is one of the richest organisms [121,122,123], and are present even in lipid fractions. The safety of conventional preservatives, such as parabens, has been revised recently by dermatological associations, and is now considered safer than a few years ago, but these AMPs could be used as novel alternative preservatives in cosmetics [124]. AMPs could be working as valuable additives for the formulation of lotions and creams as skin health promoters. Furthermore, insects are also a source of chitin and its derived chitosan that could be used in cosmetic and cosmeceutical products for skincare but even for nail, hair, and lip products, as an alternative to crustacean chitin and chitosan [125,126,127].

The scientific and economic literature on this topic is very limited. Despite that, the possibility to use lipids from insects, especially if we consider bioconverter insects, integrated into a circular economy framework, is a good opportunity to fully exploit their potentiality.

## 5. Conclusions and Future Perspectives

Lipids from insects are a suitable perspective for personal care product formulations. To date, different lipids extraction methods are exploited at a laboratory scale. It is necessary to use industrial extraction and purification methods in a sustainable way. Natural substances have recently made a comeback, making the industrial sector more ecologically friendly. In this context, insects could show numerous advantages. Insects have a strong environmental adaptation ability, so they do not compete with crops for food production purposes, they require less water, and they have a high biomass production in favorable conditions of rearing and show high-fat contents, similar to coconut oil and palm kernel oil production. Moreover, the production of CO_2_ emissions required by their breeding is much less than crop cultivation. In regard to the many advantages associated with lipids production, the possibility to manage the fatty acid profile of insects through the feeding substrates is the most interesting. Moreover, insect lipids can be used as a source for cosmetic products with the application depending on the species. The cosmetics industry is always in search of new ingredients to use in novel product formulations. Furthermore, the processing of insect lipids, such as the extraction and purification steps, is often complex and expensive. Although there are still numerous issues to be resolved in order to extend the production of insects on a large scale, they are a promising sustainable resource for lipids and many other bioactive molecule productions such as proteins, chitin, chitosan, and AMPs [121,122,123,126,127]. To make insect industrial utilization economically sustainable, lipids production from insects can be associated with other high-value products, specifically with insect proteins. Moreover, lipids can be co-products of environmental applications of bioconverter insects, for example, for the management of organic waste in an unconventional and innovative way [128,129].

## Figures and Tables

**Table 1:** Lipid yield and fatty acid compositions in seven insect species that could be to be reared for feed production for aquaculture, poultry, and pig farming according to European Regulations 873/2017 and 2021/1372 and some plant oil. DW = dry weight.

	Lipid Yield (%/DW)	C 12:0 (%)	C 14:0 (%)	C 16:0 (%)	C18:1 n-9 (%)
*Hermetia illucens* [46]	7.20–42.60	23.90–61.87	3.85–10.35	5.78–20.42	4.27–28.80
*Musca domestica* [47]	21.70 ± 1.70	0.00	0.46 ± 0.01	5.10 ± 0.07	7.21 ± 0.08
*Tenebrio molitor* [48]	31.97 ± 1.60	0.00	4.45 ± 0.02	21.33 ± 0.13	35.83 ± 0.33
*Alphitobius* *diaperinus* [49]	26.25 ± 0.01	0.03 ± 0.04	0.77 ± 0.01	24.98 ± 0.05	28.97 ± 0.01
*Acheta domesticus* [48]	15.31 ± 0.18	0.1 ± 0.00	0.44 ± 0.00	22.65 ± 0.37	20.18 ± 0.02
*Gryllodes sigillatus* [50]	18.23 ± 0.70	0.1 ± 0.02	1.65 ± 0.12	23.5 ± 0.65	29.14 ± 1.50 (C18:1n9c + C18:1n9)
*Gryllus assimilis* [51]	21.80 ± 2.65	0.10 ± 0.02	1.00 ± 0.00	33.10 ± 1.86	30.30 ± 0.27
Coconut oil [52,53]	33.00	44.10–51.00	13.10–18.50	7.50–10.50	5.00–8.20
Commercial palm oil [54,56]	45.00–55.00	0.20	1.10	44.00	39.20
Commercial palm kernel oil [54,55]	50.00	47.80	16.30	8.50	15.40

**Table 2:** Lipids yield and related extraction methods in the seven insect species admitted by European Regulations 893/2017 and 1372/2021. DW = dry weight, WW = wet weight.

Insect Species	Lipid Yield (%)	Extraction Methods
*Hermetia illucens*	40.96 ± 0.93 (DW)	Soxhlet (Petroleum ether) [59]
	34.54 (WW)	Folch [60]
	37.10 ± 1.10 (DW)	Soxhlet (Diethyl ether) [11]
*Musca domestica*	21.7 ± 1.7 (DW)	Soxhlet (Petroleum ether) [47]
	24.56 (DW)	Soxhlet (Petroleum ether) [61]
*Tenebrio molitor*	7.8 ± 0.4 (WW)	Aqueous [57]
	12.7 ± 2.4 (WW)	Soxhlet [57]
	12.9 ± 0.2 (WW)	Folch [57]
	25.5 ± 0.1 (WW)	Soxhlet (Hexane) [58]
	24.3 ± 1.2 (WW)	Soxhlet (Petroleum ether) [58]
	25.7 ± 0.3 (WW)	Soxhlet (Ethyl acetate) [58]
	28.8 ± 5.9 (WW)	Soxhlet (Ethanol) [58]
	23.7 ± 2.4 (WW)	TPP [58]
	22.1 ± 0.6 (WW)	SC-C2 [58]
*Alphitobius diaperinus*	5.5 ± 1.0 (WW)	Aqueous [57]
	10.7 ± 0.5 (WW)	Soxhlet [57]
	9.4 ± 1.0 (WW)	Folch [57]
*Acheta domesticus*	1.6 ± 0.1 (WW)	Aqueous [57]
	6.0 ± 0.3 (WW)	Soxhlet [57]
	8.0 ± 1.1 (WW)	Folch [57]
	14.6 ± 0.1 (WW)	Soxhlet (Hexane) [58]
	14.7 ± 0.2 (WW)	Soxhlet (Petroleum ether) [58]
	15.1 ± 0.3 (DW)	Soxhlet (Ethyl acetate) [58]
	22.7 ± 2.9 (DW)	Soxhlet (Ethanol) [58]
	19.3 ± 2.0 (DW)	TPP [58]
	11.9 ± 1.4 (DW)	SC-CO_2_ [58]
*Gryllodes sigillatus*	18.23 ± 0.7 (DW)	Soxhlet (hexane) [50]
*Gryllus assimilis*	21.80 ± 2.65 (DW)	Soxhlet (Petroleum ether) [51]

## Data Availability

Not applicable.
